# A phase II/III randomized clinical trial of CisPlatin plUs Gemcitabine and Nabpaclitaxel (GAP) as pReoperative chemotherapy versus immediate resection in patIents with resecTable BiliarY Tract Cancers (BTC) at high risk for recurrence: PURITY study

**DOI:** 10.1186/s12885-024-12225-6

**Published:** 2024-04-08

**Authors:** Monica Niger, Federico Nichetti, Lorenzo Fornaro, Chiara Pircher, Federica Morano, Federica Palermo, Lorenza Rimassa, Tiziana Pressiani, Rossana Berardi, Andrea Casadei Gardini, Elisa Sperti, Lisa Salvatore, Davide Melisi, Francesca Bergamo, Salvatore Siena, Stefania Mosconi, Raffaella Longarini, Giuseppina Arcangeli, Salvatore Corallo, Laura Delliponti, Stefano Tamberi, Elena Fea, Giovanni Brandi, Ilario Giovanni Rapposelli, Massimiliano Salati, Paolo Baili, Rosalba Miceli, Silva Ljevar, Ilaria Cavallo, Elisa Sottotetti, Antonia Martinetti, Michele Droz Dit Busset, Carlo Sposito, Maria Di Bartolomeo, Filippo Pietrantonio, Filippo de Braud, Vincenzo Mazzaferro

**Affiliations:** 1 Department of Medical Oncology, Fondazione IRCCS Istituto Nazionale dei Tumori, Via G. Venezian, 1, 20133 Milan, Italy; 2 Computational Oncology, Molecular Diagnostics Program, National Center for Tumor Diseases (NCT) and German Cancer Research Center (DKFZ), Heidelberg, Germany; 3 Medical Oncology Unit, Azienda Ospedaliero-Universitaria Pisana, Pisa, Italy; 4 Department of Biomedical Sciences, Humanitas University, Pieve Emanuele (Milan), Italy; 5 Medical Oncology and Hematology Unit, Humanitas Cancer Center, IRCCS Humanitas Research Hospital, Rozzano (Milan), Italy; 6 Clinica Di Oncologia Medica, A.O.U. Delle Marche, Università Politecnica Delle Marche, Ancona, Italy; 7 Vita-Salute San Raffaele University, Milan, Italy; 8 Department of Medical Oncology, San Raffaele Scientific Institute IRCCS, Milan, Italy; 9 Department of Oncology, University of Turin, AO Ordine Mauriziano Hospital, Turin, Italy; 10 Oncologia Medica, Comprehensive Cancer Center, Fondazione Policlinico Universitario Agostino Gemelli IRCCS, Rome, Italy; 11 Oncologia Medica, Università Cattolica del Sacro Cuore, Rome, Italy; 12 Digestive Molecular Clinical Oncology Research Unit, Università Degli Studi Di Verona, Verona, Italy; 13 Investigational Cancer Therapeutics Clinical Unit, Azienda Ospedaliera Universitaria Integrata, Verona, Italy; 14 Medical Oncology 1, Veneto Institute of Oncology IOV-IRCCS, Padua, Italy; 15 Department of Hematology Oncology, and Molecular Medicine, Grande Ospedale Metropolitano Niguarda, Milan, Italy; 16 Department of Oncology and Hemato-Oncology, University of Milan, Milan, Italy; 17 Medical Oncology Unit, Giovanni XXIII Hospital, Bergamo, Italy; 18 San Gerardo Hospital, Monza, Italy; 19 Department of Medical Oncology, ASST Spedali Civili, Brescia, Italy; 20 Medical Oncology Unit, Fondazione IRCCS Policlinico San Matteo, Pavia, Italy; 21 Department of Medical Oncology, Ospedale Santa Maria Delle Croci, Ravenna AUSL Romagna, Italy; 22 Department of Medical Oncology, S. Croce E Carle Teaching Hospital, Cuneo, Italy; 23 Medical Oncology, IRCCS Azienda Ospedaliera, Universitaria Di Bologna, Bologna, Italy; 24 Department of Medical Oncology, IRCCS Istituto Romagnolo Per Lo Studio Dei Tumori (IRST) “Dino Amadori”, Meldola, Italy; 25 Oncology Unit, University Hospital of Modena, Modena Cancer Centre, Modena, Italy; 26 Department of Epidemiology and Data Science, Data Science Unit, Fondazione IRCCS Istituto Nazionale dei Tumori, Milan, Italy; 27 Biostatistics for Clinical Research Unit, Fondazione IRCCS Istituto Nazionale dei Tumori, Milan, Italy; 28 Scientific Directorate, Fondazione IRCCS Istituto Nazionale dei Tumori, Milan, Italy; 29 Department of Surgery, Division of HPB, General Surgery and Liver Transplantation, Fondazione IRCCS Istituto Nazionale dei Tumori, Milan, Italy

**Keywords:** Biliary tract cancers, Cholangiocarcinoma, Neoadjuvant chemotherapy, Cisplatin gemcitabine nabpaclitaxel

## Abstract

**Background:**

Biliary tract cancers (BTCs) are rare and lethal cancers, with a 5-year survival inferior to 20%(1–3). The only potential curative treatment is surgical resection. However, despite complex surgical procedures that have a remarkable risk of postoperative morbidity and mortality, the 5-year survival rate after radical surgery (R0) is 20–40% and recurrence rates are up to ~ 75%(4–6). Up to ~ 40% of patients relapse within 12 months after resection, and half of these patient will recur systemically(4–6). There is no standard of care for neoadjuvant chemotherapy (NAC) in resectable BTC, but retrospective reports suggest its potential benefit (7, 8).

**Methods:**

PURITY is a no-profit, multicentre, randomized phase II/III trial aimed at evaluating the efficacy of the combination of gemcitabine, cisplatin and nabpaclitaxel (GAP) as neoadjuvant treatment in patients with resectable BTC at high risk for recurrence. Primary objective of this study is to evaluate the efficacy of neoadjuvant GAP followed by surgery as compared to upfront surgery, in terms of 12-month progression-free survival for the phase II part and of progression free survival (PFS) for the phase III study.

Key Secondary objectives are event free survival (EFS), relapse-free survival, (RFS), overall survival (OS), R0/R1/R2 resection rate, quality of life (QoL), overall response rate (ORR), resectability. Safety analyses will include toxicity rate and perioperative morbidity and mortality rate.

Exploratory studies including Next-Generation Sequencing (NGS) in archival tumor tissues and longitudinal ctDNA analysis are planned to identify potential biomarkers of primary resistance and prognosis.

**Discussion:**

Considering the poor prognosis of resected BTC experiencing early tumor recurrence and the negative prognostic impact of R1/R2 resections, PURITY study is based on the rationale that NAC may improve R0 resection rates and ultimately patients’ outcomes. Furthermore, NAC should allow early eradication of microscopic distant metastases, undetectable by imaging but already present at the time of diagnosis and avoid mortality and morbidity associated with resection for patients with rapid progression or worsening general condition during neoadjuvant therapy. The randomized PURITY study will evaluate whether patients affected by BTC at high risk from recurrence benefit from a neoadjuvant therapy with GAP regimen as compared to immediate surgery.

**Trial registration:**

PURITY is registered at ClinicalTrials.gov (NCT06037980) and EuCT(2023–503295-25–00).

## Background

Biliary tract cancers (BTCs) account for < 3% of all malignancies and include a cluster of heterogeneous tumors arising from the intrahepatic (iCCA), perihilar (pCCA) and distal (dCCA) biliary tree or from the gallbladder (GBC). BTCs are rare, but their incidence (0.3–6 per 100,000 inhabitants per year) and mortality (1–6 per 100,000 inhabitants per year, globally, except for specific regions with incidence > 6 per 100,000 habitants such as South Korea, China and Thailand) have been increasing in the past few decades worldwide, representing a global health problem.

Overall, BTCs are highly lethal with a 5-year survival ranging from 5 to 40% [1–3]. The only potential curative treatment is surgical resection. However, more than 50% of patients present with unresectable disease at diagnosis. Furthermore, despite the high surgical effort, with complex procedures that have a remarkable risk of postoperative morbidity and mortality, the 5-year survival rate after radical surgery (R0) is 20–40% and recurrence rates are up to ~ 75% in iCCA [4], ~ 60% in eCCA [9] and 50% in GBC [6]. Recent reports showed that ~ 40% of iCCA [4], ~ 30% of eCCA [9] and ~ 40% of GBC [6] relapse within 12 months after resection. Roughly 40–50% of these patients will recur systemically.

Standard of care adjuvant treatment is capecitabine for 8 cycles, as per the BILCAP trial [10]. Although BILCAP was negative for the prespecified primary endpoint [overall survival (OS) by intention to treat], the study showed that adjuvant capecitabine improved OS compared with observation in the per-protocol population, with a clinically meaningful benefit in median OS (mOS, 53 vs 36 months, HR 0.75). However, in both arms, recurrence rate was quite high (63% overall, 60% in the capecitabine group and 65% in the observation group).

There are many established risk factors for recurrences and overall worsening prognosis in resected BTCs, which have been evaluated for the development of standardized risk assessment models [11, 12]. These tools can help to identify patients at risk for adverse outcomes following tumor resection and thereby to facilitate treatment decisions. Unfortunately, the implementation of preoperative risk scores in clinical practice has been limited.

Considering the poor prognosis of resected BTC experiencing early tumor recurrence and the negative prognostic impact of R1/R2 resection, neoadjuvant chemotherapy may improve R0 resection rates and patients’ outcomes. Currently, there is no standard of care or prospective data for neoadjuvant chemotherapy (NAC) in resectable BTC, but retrospective reports suggest the potential benefit of a pre-operative treatment [7, 8, 13].

Cisplatin, gemcitabine and nab-paclitaxel (GAP) has shown to be an active regimen for advanced BTC [14], and in particular for patients affected by localized disease with a manageable safety profile [15, 16].

Based on these data, our hypothesis is that a treatment with gemcitabine, cisplatin and nabpaclitaxel as neoadjuvant chemotherapy may improve the outcome of patients with resectable BTC without an exceeding cost of toxicity.

The PURITY study aims at investigating the GAP regimen as neoadjuvant triplet chemotherapy for patients with resectable BTC harboring high risk features for postoperative recurrence, including large tumor size, multifocal disease, macrovascular invasion or positive locoregional lymph nodes at preoperative imaging and/or high levels of Ca19.9.

## Methods

### Aims

Primary objective of this study is to evaluate the efficacy of neoadjuvant GAP followed by surgery as compared to upfront surgical approach in terms of 12-month progression-free survival (PFS, phase II part) and PFS (phase III). Key Secondary objectives are event free survival (EFS), relapse-free survival, (RFS), overall survival (OS), R0/R1/R2 resection rate, quality of life (QoL), overall response rate (ORR), resectability. Safety analisys will include toxicity rate and perioperative morbidity and mortality rate.

Exploratory studies including Next-Generation Sequencing (NGS) in archival tumor tissues and longitudinal ctDNA analysis are planned in order to identify potential biomarkers of primary resistance and prognosis.

## Trial design

**Fig. 1:**
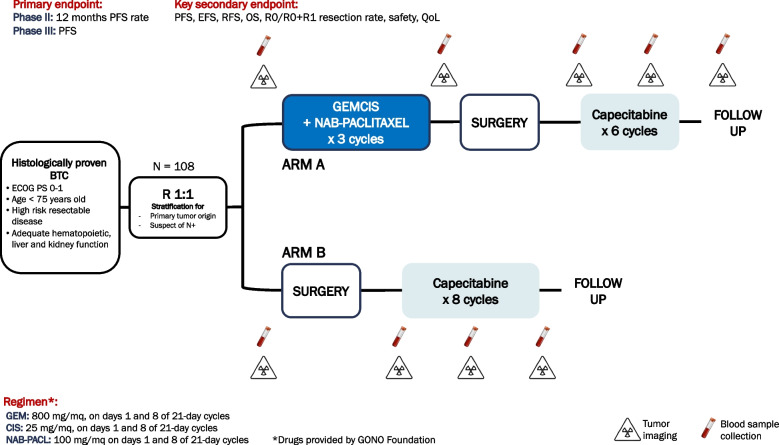
PURITY Study design

PURITY is a multicenter, randomized adaptive phase II/III trial aimed at comparing the triplet combination of gemcitabine, cisplatin and nabpaclitaxel as neoadjuvant treatment (ARM A) versus standard upfront surgery (ARM B) in terms of 12-month PFS (phase II part) and PFS (phase III part) in patients with resectable BTC at high risk for recurrence. The study design is depicted in Fig. 1.

Tumor resectability and risk factors for recurrence will be evaluated by a local multidisciplinary tumor board, including a core team with at least one medical oncologist, one surgeon, one radiologist, one endoscopist/gastroenterologist and one pathologist, all with expertise > 3 years on biliary tract cancer and hepatobiliary oncology.

Patients will be enrolled by their treating investigators and assigned to a treatment arm by 1:1 adaptive randomization via minimization. Before randomization the patients will be stratified by primary tumor origin (intrahepatic vs extrahepatic cholangiocarcinoma vs gallbladder cancer) and suspected or definite locoregional lymph nodes. The phase II of the study will enroll 108 patients; if the results of the phase II part are positive, an expansion of the sample size up to 250–300 patients overall (including patients of the phase II part) is planned for the phase III part, based on predictive power calculation. The randomization will continue with the same criteria described above. The study will be sponsored by GONO Foundation and it will be open at 19 study centers in Italy (Table 1), including the coordinating center Fondazione IRCCS Istituto Nazionale Tumori. Registration, randomization and data collection procedures will be performed using the Research Electronic Data Capture (REDCap) platform, which is a secure, browser-based web application widely used by researchers offering unique features, including the adaptive randomizazion via minimization, that can be used to conduct rigorous RCTs. Investigator meetings and monthly accrual updates will be held to ensure adequate enrollment.

**Table 1:** Participating centers

PARTICIPATING CENTERS
Fondazione IRCCS Istituto Nazionale dei Tumori di Milano
Università di Modena
Ospedali Riuniti di Ancona
Azienda Ospedaliera Universitaria Pisa
Ospedale San Raffaele Milano
Humanitas Cancer Center Milano
Azienda Ospedaliera Ordine Mauriziano di Torino
Policlinico Gemelli Roma
AOUI Verona—Policlinico "G.B. Rossi"
IOV Padova
Ospedale Niguarda Cancer Center, Milano
ASST Papa Giovanni XXIII—Bergamo
Ospedale S.Gerardo Monza
ASST Spedali Civili Brescia
Policlinico San Matteo, Pavia
Ospedale Santa Maria delle Croci—Ravenna
Azienda Ospedaliera S. Croce e Carle di Cuneo
Oncologia Medica Policlinico Sant’Orsola- Malpighi Bologna
IRCCS IRST "Dino Amadori" Meldola

The Sponsor Gruppo Oncologico Nord Ovest (GONO) will be responsible for data management of this study, including quality checking of the data.

## Study endpoints

The primary endpoint of the Phase II part of the study (that is a secondary endpoint in the phase III part of the study) is the 12-month PFS, i.e. the proportion of patients alive and free from progression/post-resection recurrence at 12-months from randomization.

The primary endpoint of phase III part of the study (that is a secondary endpoint in the phase II part of the study) is the PFS, defined as the time interval from randomization to disease progression, post-resection recurrence or death from any cause. The time will be censored on the date of the last evaluable on study tumor assessment documenting absence of progressive disease for patients who are alive, on study and progression free at the time of the analysis. Alive patients having no tumor assessments after baseline will be attributed a PFS time censored at the randomization date.

Additional secondary endpoints are:

- ▪ EFS (Event free survival), i.e. the time from randomization to disease progression that precludes definitive surgery, treatment discontinuation for any reason, post-resection recurrence, a second primary cancer, or death from any cause.
- ▪ RFS (Relapse-free survival), i.e. the time from surgery to disease recurrence or death in patients who undergo to surgery with curative intent.
- ▪ OS (overall survival), i.e. the time from randomization to death or last follow-up for alive patients.
- ▪ Overall Response Rate (ORR) of neoadjuvant therapy as per investigator assessment and central review, i.e. the percentage of patients, relative to the total of enrolled subjects receiving neoadjuvant treatment, achieving a complete (CR) or partial (PR) response, according to RECIST 1.1 criteria.
- ▪ Resectability rate of primary tumor, i.e. the retrospective evaluation of patients with unresectable disease, as assessed by the central review committee, in the two arms, and of the rate of conversion to resectability in the neoadjuvant arm.
- ▪ R0 (+ R1) resection rate, i.e. the percentage of patients, relative to the total of randomized patients, for whom the tumor was macro- (and micro-)scopically removed and the intent of surgery is considered curative.
- ▪ Toxicity, i.e. the percentage of patients, relative to the total of subjects randomized to neoadjuvant treatment, experiencing a specific adverse event, according to National Cancer Institute Common Toxicity Criteria (version 5.0).
- ▪ Perioperative morbidity and mortality, i.e. the percentage of patients, relative to the total of enrolled subjects undergoing surgery, with any serious perioperative morbidity or mortality according to Clavien-Dindo classification.
- ▪ Quality of life (QoL), i.e. QoL will be estimated with EORTC QLQ-C30 and the modules BIL21 and INFO25, PEF-FB-9 (SDM-Q-9) and PEF-FB-Doc (SDM-Q-Doc).

As exploratory endpoints, potential biomarkers and their correlation with outcome measures will be investigated as follows: multi-omic (genomic + transcriptomic) tumor profiling in tumor biopsies and in surgical samples will be explored and associated with ORR, PFS and OS in each trial arm; liquid biopsies will be performed with the aim to track circulating tumor (ct)DNA clearance induced by chemotherapy in the neoadjuvant arm, detect minimal residual disease (MRD) in post-surgical time-points in both arms and describe the clonal evolution of the disease during treatments by ultra-deep sequencing techniques; radiogenomic and radiomic analyses will be performed to predict the presence of specific molecular targets and tumor heterogeneity in all patients, as well as pathological response to preoperative treatment in the experimental arm; covariates and published preoperative risk assessment for BTC scores will be prospectively assessed and associated with PFS and OS. Samples and imaging scans will be stored at Fondazione IRCCS Istituto Nazionale Tumori.

## Clinical Setting

Patients affected by histologically or cytologically confirmed non metastatic resectable BTC can be evaluated for the study. To be considered eligible, patients must have technically resectable BTC as per local multidisciplinary team (MDT) assessment, including a core team with at least one medical oncologist, one surgeon, one radiologist, one endoscopist/gastroenterologist and one pathologist, all with expertise > 3 years on biliary tract cancer and hepatobiliary oncology.

Other main inclusion criteria are:

- Age ≥ 18 years and < 75 years
- ECOG PS 0–1
- Adequate hematologic, hepatic, renal and coagulation function
- Available archival tumor tissue for exploratory research
- Exclusion of distant metastases by CT or MRI of abdomen, pelvis, and thorax and PET scan.
- High risk for recurrence defined as the presence of at least one of the following risk features, as evaluated at baseline (pre-surgery):
  - ◦ For cholangiocarcinoma:
    - ▪ Suspected or definite locoregional lymph node involvement (at least one of the following):
    - ▪ positive FNA cytology (obtained by EUS).
    - ▪ positive locoregional lymph nodes at PET-CT.
    - ▪ suspected positive locoregional lymph nodes at imaging (CT or MRI scan) according to local MDT discussion (eg. short axis > 1.5 cm, contrast enhancement uptake, round shape, restriction at DWI).
    - ▪ Macrovascular invasion at preoperative CT scan.
    - ▪ Expected R1 resection due to proximity to major intrahepatic vascular and biliary structures.
    - ▪ For iCCA, presence of satellitosis or multifocal disease or radiological suspicion of tumoral diaphragmatic adhesion.
    - ▪ For iCCA, size of the liver lesion > 5 cm.
    - ▪ For eCCA, size of the primary lesion > 3 cm.
    - ▪ Ca19.9 > 100 U/mL.
  - ◦ For GBC:
    - ▪ Incidentally Detected Gallbladder Carcinoma (IGBC) after simple cholecystectomy with indication for radical second surgery (> pT2) or newly diagnosed GBC.

Main exclusion criteria are:

- Locally unresectable tumor according to local MDT (including radiological evidence suggesting inability to resect with curative intent whilst maintaining adequate vascular inflow and outflow, and sufficient future liver remnant).
- Evidence of distant metastases at any site.
- Tumors requiring multi-step surgical procedures such as two-stage hepatectomy or Associating Liver Partition and Portal vein Ligation for Staged hepatectomy (ALPPS) due to liver volumetry-based assessment of anticipated inadequate future liver remnant.
- Cirrhosis at a level of Child–Pugh B (or worse) or cirrhosis (any degree) and a history of hepatic decompensation in the year before enrolment.

## Treatment

Eligible patients will be randomized to receive:

- Arm A: GAP
  - ◦ Nab-paclitaxel 100 mg/mq, followed by.
  - ◦ Cisplatin 25 mg/mq, followed by.
  - ◦ Gemcitabine 800 mg/mq

on day 1 and 8 of 21-day cycles, for 3 cycles and subsequent surgical resection.

- Arm B: upfront surgical resection as per standard of care.

Toxicities will be evaluated following CTCAEv5.0 guidelines and managed in accordance with protocol guidelines. In ARM A, if toxicity or patient wish requires a cycle delay of more than 3 weeks, the patient should be taken off protocol treatment and undergo surgery, if feasible. To prevent and/or treat neutropenia, granulocyte-colony stimulating factors (G-CSF) administration is recommended (though not mandatory), and should be administered 24–48 h after the Day 8 administration of each treatment cycle.

Subjects with progressive disease (PD) and unresectable disease at the end of the neoadjuvant treatment will be discontinued from the study and will receive the optimal standard of care according to local guidelines and local MDT recommendation.

If resectability is confirmed, the subject will undergo tumor resection from week 10 to 12 from treatment start.

After surgery, patients in both arms will undergo adjuvant therapy with capecitabine as per standard of care, for a total of 6 cycles in ARM A and 8 cycles in ARM B, up to a total of 6 months of therapy.

The assessment of dihydropyrimidine dehydrogenase (DPYD) polymorphisms is recommended in patients who are candidates to receive fluropyrimidine according to the manufacturer's guidelines. If DPYD polymorphisms related to a deficit in the activity of the DPYD was found, the dose of 5-fluorouracil and capecitabine will be reduced according to the Italian Recommendation of Pharmacogenetics.

Adjuvant therapy will be administered starting 6–16 weeks post-surgery. Patients with R1 resection will be evaluated for adjuvant radiotherapy as per standard of care.

## Statistical methods

### Sample size justification

We plan to enroll up to 300 patients in a 1:1 randomization. In detail, for the phase II, 108 patients are required to have a 80% power to detect an increase in the primary outcome measure (12-month PFS rate) from 40% in the control group to 60% in the experimental arm, with one sided alpha held at the 10% level. If the results of the phase II part are positive, an expansion of the sample size up to 250–300 patients overall (including patients of the phase II part) is planned for the phase III part, based on predictive power calculation. We estimated that a two-sided logrank test with an overall sample size of 274 subjects (137 in the control group and 137 in the experimental group) achieves 80.0% power at a 5% significance level to detect a hazard ratio of 0.70. This prediction, however, depends on a number of assumptions that may not be fully satisfied. For this reason, a number of nuisance parameters (mainly the pattern of accrual and the event rate in the control arm) will be estimated at the end of the phase II study and the sample size to be reached for phase III will be fine-tuned accordingly with a substantial modification of the protocol, with capping at a maximum of 300 patients overall.

### Statistical analyses

Demographic and baseline characteristics such as age, sex, and baseline disease characteristics will be summarized by treatment arm for the ITT population. Descriptive baseline summaries of continuous data will present mean, standard deviation, median, minimum, and maximum. Descriptive summaries of discrete data will present the category counts as frequencies and percentages.

As for the Phase II main endpoint analysis, the proportion of patients alive and free from progression/post-resection recurrence at 12-months from randomization will be compared between the two study arms with Pearson’s Chi square test. If achieving a positive phase II result (10% significant increase of the proportion from 40% in the control group to 60% in the experimental arm), an expansion to a phase III part is planned, with PFS as primary endpoint. Kaplan Meier PFS curves will be estimated for each trial arm and the two curves will be compared by means of the logrank test. An additional analysis will be conducted by mean of the Cox proportional hazard regression model, incorporating information on recognized prognostic factors so as to obtained an adjusted estimate of experimental treatment effect. Similar analyses will be conducted on OS.

Safety analyses will be conducted on the safety population of the experimental arm, i.e. the ITT population excluding patients not receiving at least one dose of study drug. All safety parameters will be analyzed and presented in terms of listings and summary tables; adverse events will be classified according to the National Cancer Institute Common Toxicity Criteria (version 5.0). The assessment of safety will be based mainly on the frequency and nature of severe (G3/G4) AEs or serious adverse events (SAEs). Number and percentage of patients having any severe AEs or SAEs as well as the system/organ class involved in the severe AE will be presented. Any other information collected (e.g. severity or suspected relationship to study medication) will be listed as appropriate. Drug-discontinuing AEs will be closely monitored throughout the study in order to promptly close study in case of unacceptable toxicity. No formal safety interim analyses are planned for this study. Periodic safety reviews will be conducted and any outcome affecting the study conduct will be promptly communicated by the investigators for notification to the Institutional Review Boards (IRBs)/Ethics Committees (ECs). Sequential boundaries will be used to monitor unacceptable toxicity rate during the Phase II part of the trial. The accrual will be halted if excessive numbers of unacceptable toxicities are seen, that is, if this number is equal to or exceeds TN out of N patients with full follow-up (see the table below). This is a Pocock-type stopping boundary that yields at most a 10% probability of crossing the boundary (erroneous probability of early stopping) when the rate of unacceptable toxicity does not exceed the maximum acceptable rate [event probability] of 20%.

The analysis of patients’ reported outcome (PRO- assessed using the EORTC QLQ-C30, the EORTC QLQ-OG25 and the EuroQol EQ-5D questionnaires) will be performed according to the EORTC Scoring and Reference Values Manual. All scores and subscales will be assessed through descriptive summary statistics. Mean score changes from baseline, proportion of patients with improved, stable, or deteriorated scores from baseline and time to deterioration in the EORTC QLQ-C30 and BIL21 physical functioning, social functioning, and fatigue scores will be compared between the two arms.

## Discussion

PURITY is the first randomized trial assessing the impact of GAP as neoadjuvant therapy in patients affected by BTC with high risk for disease recurrence. As described above, there is a strong rationale for the use of neoadjuvant therapy to treat these patients, as the majority of them recurs even after radical surgery, and very often within the first year after surgery. Regarding the study design, ethical and pragmatic considerations are required. The introduction of a preoperative regimen in those patients who are about to undergo a curative surgery may not be safe and it is crucial to avoid compromising this type of surgery with significant delays. However, the clinical practice at referral Centers is to discuss the treatment strategy of these patients in a multidisciplinary setting and to discuss with patients the availability of clinical trials, including neoadjuvant trials, at the time of diagnosis, thus allowing to rapidly optimize patients’ management and care and avoid unnecessary delays of surgery. Safety-wise, the GAP regimen has been extensively studied and safety is both acceptable and manageable. A phase II trial was designed to test the benefit of adding nab-paclitaxel to the standard first-line regimen of gemcitabine plus cisplatin (GAP regimen) in advanced BTCs. Among 60 patients with advanced BTC, the combination of gemcitabine, cisplatin and nabpaclitaxel as a first line treatment achieved a 45% ORR and 84% disease control rate (DCR), with median PFS of 11.8 (95% CI, 6.0 to 15.6) months and median OS of 19.2 months (95% CI, 13.2 months to not estimable). Notable, 12 patients (20%) were converted from unresectable to resectable disease, and they subsequently underwent surgery [14]. More recently, results from SWOG 1815, a randomized, open-label, phase 3 trial comparing GAP to Cisplatin + Gemcitabine (CisGem) as first line treatment in patients with advanced BTC, were presented at the 2023 ASCO Gastrointestinal Cancers Symposium [15]. Overall, SWOG 1815 failed to show a significant benefit of GAP for patients with unresectable/metastatic disease. However, it is worth noticing that in an exploratory subgroup analysis of patients with locally advanced disease, the median OS was 19.2 months with GAP versus 13.7 months with CisGem (*P* = 0.01). A similar trend in PFS was seen in patients with locally advanced disease, with a median PFS of 9.3 months with GAP versus 7.6 months withCisGem (*P* = 0.04). These results confirm the rationale of the GAP regimen for localized disease and they add up to two other studies on the clinical feasibility of curative surgery after treatment with GAP regimen in patients with localized disease. First, a retrospective analysis including 129 patients affected by locally advanced CCA treated with induction GAP in South Korea [17], with a ORR and DCR in patients with measurable disease of 60.8% and 91.9%, respectively; 77 (59.7%) patients were determined as resectable after induction chemotherapy and of the 73 who actually underwent surgery, R0 resection was achieved in 67 (91.8%) and 6 (8.2%) had a complete pathological remission in the final pathology.

Then, Maithel et al. published the results of the NEO-GAP study [18], a single-arm phase II trial for patients with resectable high-risk CCA. Patients were administered 4 cycles (3 months) of preoperative GAP prior to an attempt at curative-intent surgical resection. The study met its primary endpoint and demonstrated that neoadjuvant gemcitabine/cisplatin/nab-paclitaxel is feasible and safe prior to resection of intrahepatic cholangiocarcinoma and does not adversely impact perioperative outcomes.

Most importantly, since patients with resectable BTC that will be selected for PURITY study have a unacceptably high rate of early relapse after surgery and overall mortality > 80%, neoadjuvant therapy has several potential advantages, in terms of: 1) improving the probability of radical surgery, early treatment of micrometastatic disease and, ultimately, improvement of survival; and 2) avoiding mortality and morbidity associated with resection for patients with rapid progression. These possible advantages overweight the possible risks of the neoadjuvant treatment. Based on this rationale and the data presented above, the PURITY study is aimed at evaluating in a randomized setting the actual impact of neoadjuvant GAP in patients at high risk for recurrence.

Concerning the statistical plan, the phase 2 part of the study was designed with an expected 12-month PFS of 40% in the control arm, which may be considered low. Recent findings have shown that roughly 40% of iCCA, 30% of eCCA, and 40% of GBC patients experience relapse within 12 months post-resection overall [4–6, 12]. Similarly, in BILCAP [19] the median relapse free survival was at ~ 24 months in the experimental arm. However, in this trial patients were highly selected, as successfully undergoing surgery and fit to receive adjuvant chemotherapy. In PURITY, it must be considered that patients will be eligible if they present characteristics of high risk of recurrence, which are therefore associated with worse relapse estimates than those reported above. Additionally, patients will be randomized prior to surgery, which may result in lower PFS due to patients who experience disease progression or other complications in the preoperative phase in both study arms, preventing from curative surgery. The accuracy of this 12-month PFS prediction so as to correctly interpret the results and possibly continue with the phase 3.

Finally, PFS was chosen as the primary endpoint for the phase 3, due to its potential as a surrogate for OS, considering early tumor relapse's association with poorer OS [12] and to maximize the trial’s feasibility. However, OS remains the ultimate endpoint to consider the trial as meaningfully positive, and its adoption will be adaptively discussed in light of phase 2 part results. Overall, the phase 2–3 design of the PURITY trial is per se a go-no go strategy adopted to avoid the futile overtreatments in excessively large patients’ populations. Close oversight of study conduct and of safety will be provided through contact with participating investigators and the study will be prematurely terminated if an unexpected, unacceptable risk to patients is seen.

## Data Availability

No datasets were generated or analysed during the current study.

## Abbreviations

AEs: AdverseEvent
BTC: Biliary Tract Cancer
ip/d/eCCA: Intrahepatic/Perihilar/Distal/Extrahepatic Cholangiocarcinoma
CR: Complete Response
CT: Computed Tomography
ctDNA: Circulating Tumor DNA
ECOG PS: Easter Cooperative Oncology Group Performance Status
EFS: Event Free Survival
EORTC: European Organisation For Research And Treatment Of Cancer
EUS: Endoscopic Ultrasound
GAP: Gemcitabine, Cisplatin And Nabpaclitaxel
IGBC: Incidentally Detected Gallbladder Carcinoma
MRI: Magnetic Resonance
MDT: MultidisciplinaryTeam
NAC: Neoadjuvant Chemotherapy
NGS: Next-Generation Sequencing
OS: Overall Survival
PD: Progressive Disease
PET: Positron Emission Tomography
PFS: Progression Free Survival
PR: Partial response
RFS: Relapse Free Survival
SAE: Serious Adverse Event
QoL: Quality of life
ORR: Overall response rate
