# Correction to: A phase II/III randomized clinical trial of CisPlatin plUs Gemcitabine and Nabpaclitaxel (GAP) as pReoperative chemotherapy versus immediate resection in patIents with resecTable BiliarY tract cancers (BTC) at high risk for recurrence: PURITY study

**DOI:** 10.1186/s12885-024-12328-0

**Published:** 2024-05-06

**Authors:** Monica Niger, Federico Nichetti, Lorenzo Fornaro, Chiara Pircher, Federica Morano, Federica Palermo, Lorenza Rimassa, Tiziana Pressiani, Rossana Berardi, Andrea Casadei Gardini, Elisa Sperti, Lisa Salvatore, Davide Melisi, Francesca Bergamo, Salvatore Siena, Stefania Mosconi, Rafaella Longarini, Giuseppina Arcangeli, Salvatore Corallo, Laura Delliponti, Stefano Tamberi, Elena Fea, Giovanni Brandi, Ilario Giovanni Rapposelli, Massimiliano Salati, Paolo Baili, Rosalba Miceli, Silva Ljevar, Ilaria Cavallo, Elisa Sottotetti, Antonia Martinetti, Michele Droz Dit Busset, Carlo Sposito, Maria Di Bartolomeo, Filippo Pietrantonio, Filippo de Braud, Vincenzo Mazzaferro

**Affiliations:** 1 Department of Medical Oncology, Fondazione IRCCS Istituto Nazionale dei Tumori, Via G. Venezian, 1, Milan, 20133 Italy; 2 Computational Oncology, Molecular Diagnostics Program, National Center for Tumor Diseases (NCT) and German Cancer Research Center (DKFZ), Heidelberg, Germany; 3 Medical Oncology Unit, Azienda Ospedaliero-Universitaria Pisana, Pisa, Italy; 4 Department of Biomedical Sciences, Pieve Emanuele (Milan), Humanitas University, Milan, Italy; 5 Medical Oncology and Hematology Unit, Humanitas Cancer Center, IRCCS Humanitas Research Hospital, Rozzano, Milan Italy; 6 Clinica Di Oncologia Medica, A.O.U. Delle Marche, Università Politecnica Delle Marche, Ancona, Italy; 7 Vita-Salute San Rafaele University, Milan, Italy; 8 Department of Medical Oncology, San Rafaele Scientifc Institute IRCCS, Milan, Italy; 9 Department of Oncology, University of Turin, AO Ordine Mauriziano Hospital, Turin, Italy; 10 Oncologia Medica, Comprehensive Cancer Center, Fondazione Policlinico Universitario Agostino Gemelli IRCCS, 11Oncologia Medica, Rome, Italy; 11 Oncologia Medica, Università Cattolica del Sacro Cuore, Rome, Italy; 12 Molecular Clinical Oncology Research Unit, Università Degli Studi Di Verona, Verona, Italy; 13 Investigational Cancer Therapeutics Clinical Unit, Azienda Ospedaliera Universitaria Integrata, Verona, Italy; 14 Medical Oncology 1 Veneto Institute of Oncology IOV-IRCCS, Padua, Italy; 15 Department of Hematology Oncology, and Molecular Medicine, Grande Ospedale Metropolitano Niguarda, Milan, Italy; 16 Department of Oncology and Hemato-Oncology, University of Milan, Milan, Italy; 17 Medical Oncology Unit, Giovanni XXIII Hospital, Bergamo, Italy; 18 San Gerardo Hospital, Monza, Italy; 19 Department of Medical Oncology, ASST Spedali Civili, Brescia, Italy; 20 Medical Oncology Unit, Fondazione IRCCS Policlinico San Matteo, Pavia, Italy; 21 Department of Medical Oncology, Ospedale Santa Maria Delle Croci, Ravenna AUSL Romagna, Ravenna, Italy; 22 Department of Medical Oncology, S. Croce E Carle Teaching Hospital, Cuneo, Italy; 23 Medical Oncology, IRCCS Azienda Ospedaliera, Universitaria Di Bologna, Bologna, Italy; 24 Department of Medical Oncology, IRCCS Istituto Romagnolo Per Lo Studio Dei Tumori (IRST) “Dino Amadori”, Meldola, Italy; 25 Oncology Unit, University Hospital of Modena, Modena Cancer Centre, Modena, Italy; 26 Department of Epidemiology and Data Science, Data Science Unit, Fondazione IRCCS Istituto Nazionale dei Tumori, Milan, Italy; 27 Biostatistics for Clinical Research Unit, Fondazione IRCCS Istituto Nazionale dei Tumori, Milan, Italy; 28 Scientifc Directorate, Fondazione IRCCS Istituto Nazionale dei Tumori, Milan, Italy; 29 Department of Surgery, Division of HPB, General Surgery and Liver Transplantation, Fondazione IRCCS Istituto Nazionale dei Tumori, Milan, Italy

**Correction to: BMC Cancer 24, 436 (2024)**

10.1186/s12885-024-12225-6.

Following publication of the original article [1], the authors reported an error in the funding section of their article. The correct funding statement is:

The present study is an investigator-driven trial, carried out by participating clinicians, who have the intellectual ownership of the results. The study is sponsored by Gruppo Oncologico del Nord Ovest, Cooperative Group having its headquarters at Via G. Mameli, 3–16,122 Genoa (ITALY) and funded by GONO Foundation. Part of the funding is also provided by Fondazione IRCCS Istituto Nazionale dei Tumori di Milano, through institutional funds and a nonconditional support of Fondazione Anna Villa e Felice Rusconi Onlus. This project is also in collaboration with Intesa Sanpaolo S.p.A. No funds can be provided to ethical committees and single participating centers. The study will be conducted according to the current regulations.
